# High intake of ultra-processed food is associated with dementia in adults: a systematic review and meta-analysis of observational studies

**DOI:** 10.1007/s00415-023-12033-1

**Published:** 2023-10-13

**Authors:** Alex E. Henney, Conor S. Gillespie, Uazman Alam, Theresa J. Hydes, Clare E. Mackay, Daniel J. Cuthbertson

**Affiliations:** 1 Department of Cardiovascular & Metabolic Medicine, University of Liverpool, Liverpool, UK; 2 Metabolism and Nutrition Research Group, Liverpool University Hospitals NHS Foundation Trust, Liverpool, Merseyside UK; 3 Department of Clinical Neurosciences, University of Cambridge, Cambridge, UK; 4 Department of Gastroenterology and Hepatology, Liverpool University Hospitals NHS Foundation Trust, Liverpool, UK; 5 Department of Psychiatry, University of Oxford, Oxford, UK; 6 Aintree University Hospital, Liverpool, UK

**Keywords:** Ultra-processed food, NOVA, Dementia, Metabolic syndrome, Alzheimer’s dementia, Mild cognitive impairment

## Abstract

**Background and aims:**

Poor cardiometabolic health is associated with dementia. Considering previous meta-analyses have confirmed associations between ultra-processed foods (UPFs) and cardiometabolic disease, we were interested in the contribution of UPF consumption to the risk of developing dementia.

**Methods:**

We performed a systematic review and meta-analysis of all records registered on Ovid Medline and Web of Science from inception until December 2022 [PROSPERO (CRD42023388363)]. Studies that assessed UPF consumption in adults, determined according to NOVA, and that reported dementia (Alzheimer’s disease, vascular dementia and mild cognitive impairment) determined by clearly stated diagnostic criteria (including formal assessment of dementia or use of diagnostic codes) were included. The association between UPF consumption and dementia was assessed using random-effects meta-analysis, controlling for confounding variables. Study quality was assessed using the Newcastle Ottawa Scale and evidence credibility evaluated using the NutriGrade system.

**Results:**

Seven thousand ten records were screened, and 122 records underwent full text review. From these, 10 observational (8 longitudinal) studies, analysing 867,316 individuals, were included. Included studies adjusted for age, socioeconomic status and co-morbidity, alongside other confounders. *High* (*vs.* low) intake of UPF was associated with increased risk of dementia (pooled relative risk 1.44 (95% confidence interval 1.09–1.90) (p = 0.02)) (I^2^ = 97.0%), although m*oderate* (*vs.* low) intake of UPF was not (1.12 (0.96–1.31) (0.13)) (85.0%). Funnel plots demonstrate low risk of publication bias.

**Conclusion:**

High UPF consumption is associated with dementia. Public health measures to reduce overconsumption of UPFs are imperative to reduce the burden of dementia.

**Graphical abstract:**

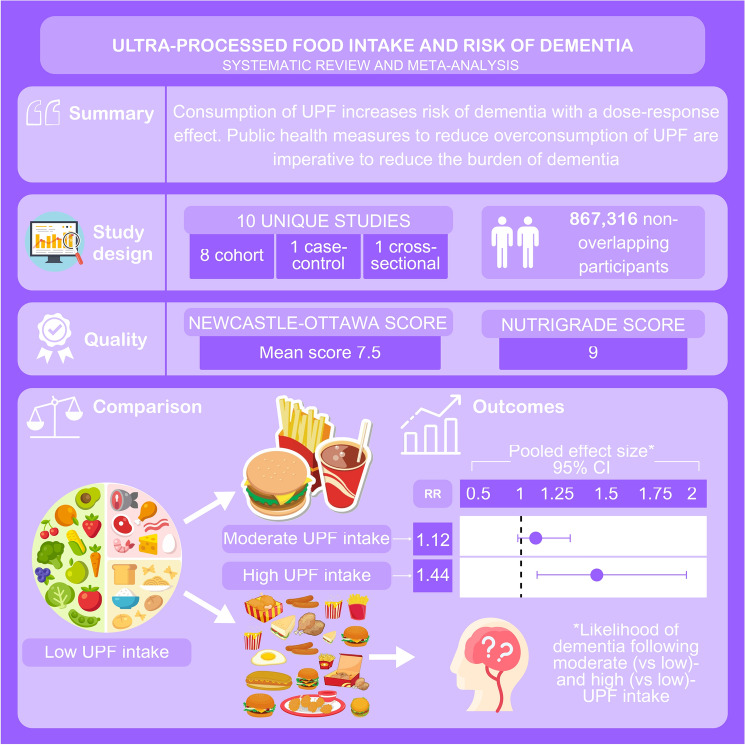

**Supplementary Information:**

The online version contains supplementary material available at 10.1007/s00415-023-12033-1.

## Introduction

Dementia is currently recognised as an umbrella term for a number of neurological conditions, of which the major symptom is the decline in brain function due to physical changes in the brain [1]. 60–70% of cases are attributed to Alzheimer’s disease (AD), although other subtypes exist including vascular (VD), frontotemporal and Lewy body dementia [2]. Owing to ageing populations, dementia prevalence is increasing. It is now the most common ageing-related disease, with over 55 million cases reported worldwide [3]. With the incidence rate of dementia at 10 million, it is estimated that 140 million people will be living with dementia by 2050 [3]. At present, there is no cure for dementia, and as such research prioritises disease prevention and retarding progression. With ~ 35% of dementia attributable to modifiable risk factors [4], the association between metabolic disease and dementia is emerging. Dementia risk increases with every additional component of the metabolic syndrome [5]. In addition, socioeconomic status (SES), strongly associated with metabolic disease [6], also increases dementia risk [7], suggesting that health inequalities contribute to increasing dementia prevalence [8].

The substantial transformation of global food systems, with accelerating ultra-processed food (UPF) production and consumption, provides fertile ground for widening health inequalities along the socioeconomic gradient [9]. UPFs are manufactured foods, such as confectionary sweets, sugar sweetened beverages (SSBs) and packaged ‘ready-meals’, formulated from by-products of high yield crops and remnants of intense animal agriculture and characterised by high energy–density with poor nutritional profiles [10]. UPFs were designed to be convenient, enjoyable and cheaper than minimally processed foods; as such they contribute ~ 50% of total energy intake (TEI) in the western world, increasing to 70% in children and lower income households [11, 12]. NOVA is the full and proper name of a tool used to stratify food products to one of four groups based on processing-related criteria (Supplementary Table S1). It has been deemed the most comprehensive classification system for UPFs [13].

To date, meta-analyses demonstrate associations between diet quality and dementia, with adherence to a Mediterranean diet being protective [14, 15], as well as convincingly demonstrating associations between UPF and non-communicable diseases including obesity, type 2 diabetes (T2D), fatty liver disease (NAFLD) and cardiovascular disease (CVD) [16–20]. Moreover, experimental studies highlight associations between UPFs and cognitive decline [21–23]. Therefore, it is imperative to objectively review the association between UPFs and dementia through meta-analysis, whilst accounting for confounding variables such as age, SES and co-morbidity that independently increase dementia risk [3].

Thus, the primary aim of the current review is to assess and quantify the relationship between UPF consumption and dementia prevalence, through systematic review and meta-analysis. The secondary aims are to assess and quantify the relationship between UPFs and dementia subtypes, and to determine whether a dose–response relationship exists between UPFs and dementia.

## Methods

The protocol for this review was registered on PROSPERO (CRD42023388363).

### Search strategy and selection criteria

The Preferred Reporting Items of Systematic Reviews and Meta-Analyses guidelines were used to construct this review [24]. Medline and Web of Science were searched (AH and CG) on 24.12.2022 for all original research describing associations between UPF and dementia. The search algorithm used was comprised of two groups of keywords described in Supplementary Table 2, with the Boolean operator ‘OR’ separating words within groups, and ‘AND’ being used between groups. We also performed manual searches of reference lists of relevant studies and contacted experts in the field to identify additional articles. No restriction was placed on the earliest search date.

### Selection criteria

To be included in our study, the criteria highlighted in Supplementary Table S3 were applied. We included MCI in our definition of dementia as per previous meta-analyses which have studied the relationship between diet with dementia [14, 15]. NOVA assigns food to one of four groups: (NOVA1) unprocessed or minimally processed foods; (NOVA2) culinary ingredients; (NOVA3) processed foods; and (NOVA4) ultra-processed foods [13]. A more detailed breakdown of what foods make up these four groups can be found in Supplementary Table S1. To be selected, studies needed to define foods as being in NOVA4 using the NOVA classification tool, or, alternatively, provide enough information on the foods consumed to enable us to determine their NOVA group.

### Outcome

The *primary outcome* of our study was the difference in dementia prevalence between patients with low *vs.* high UPF intake. Low UPF intake, defined as the non- or lowest consumption, was our reference group. High UPF intake was defined as the highest value. The *secondary outcomes* were (1) the difference in prevalence of dementia subtypes between patients with low *vs.* high intake of UPF, and (2) assessment of a dose–response association between UPF consumption and dementia. Secondary outcome 1 was assessed by stratifying dementia into all-cause dementia and the dementia subtypes, whilst outcome 2 was assessed by the difference in dementia prevalence in patients with a low *vs* moderate and high UPF intake. We considered moderate UPF intake to be the group after the reference group; in other words, the first exposure group (most commonly the second quartile or tertile of intake). The exception to this was if intake was stratified by quintiles, in which case the third quintile was used.

### Study selection

Two reviewers (AH and CG) used the selection criteria to identify appropriate literature from databases; using Rayyan to navigate the selection process. Articles were screened by titles and abstract before full texts of selected articles were reviewed. Disagreements were resolved via discussion between the two reviewers.

### Data extraction

The following data was extracted by reviewer one (AH) and independently checked by reviewer two (CG): (1) basic study information (author name, year of publication, journal) and (2) study design, population, country, study type, sample size, follow up, adjustment for confounders, definitions for UPF and dementia (dietary assessment tool, use of NOVA, dementia diagnostic tool), study outcomes (reported risk estimates in relation to dementia development). For studies that did not report the necessary data, corresponding authors were contacted.

### Quality of evidence

The Newcastle Ottawa scale (NOS) [25] was utilised to assess evidence quality. NOS is a validated tool recommended by Cochrane to assess observational research. It is composed of eight items that evaluate study selection, comparability and outcome, with a maximum score of nine. We stratified evidence into three groups: low quality scored < 5 stars, medium quality scored 5 or 6 stars and high-quality scored > 6 stars [16]. In addition, the NutriGrade scoring system was used to assess evidence credibility. The tool is an eight-item scale that evaluates evidence for meta-analyses related to nutrition. To interpret NutriGrade evaluation, the following scoring system was used: (a) very low (0–3.99); (b) low (4–5.99); (c) moderate (6–7.99); (d) high (8–10) [26].

### Meta-analysis

A random-effects model was used to calculate a pooled relative risk (RR) ± 95% confidence interval (CI) because data was expected to be highly heterogenous. The random effects model used was Cochran-Mantel-Haenzel test [27]. This was perfomed using R Studio V4.0.2 (R Studio PBC, USA, meta, ggplot2, and metafor packages). The Higgins I^2^ statistical technique was used to assess heterogeneity [28], supported by the Paule-Mandel test to estimate tau^2^ [29]. To be considered highly heterogenous, values were required to be > 75% (p =  < 0.05). Thereafter, summary level meta-regression analysis was performed using a fixed-effects model to evaluate whether a dose–response association existed between increasing UPF intake and risk of dementia (metareg and bubble functions from meta package).

Sensitivity analyses assessed the impact of the following variables: study design, reporting of UPF (NOVA or non-NOVA classified), sample size, continent, type of dementia, and study quality. A further series of analyses were performed, based on adjustment for confounders, to strengthen any association between UPF and dementia. We used the result from the fully adjusted model in the included studies when conducting all analyses. Begg’s funnel plots were generated for visualisation of publication bias.

## Results

### Study characteristics

**Fig. 1:**
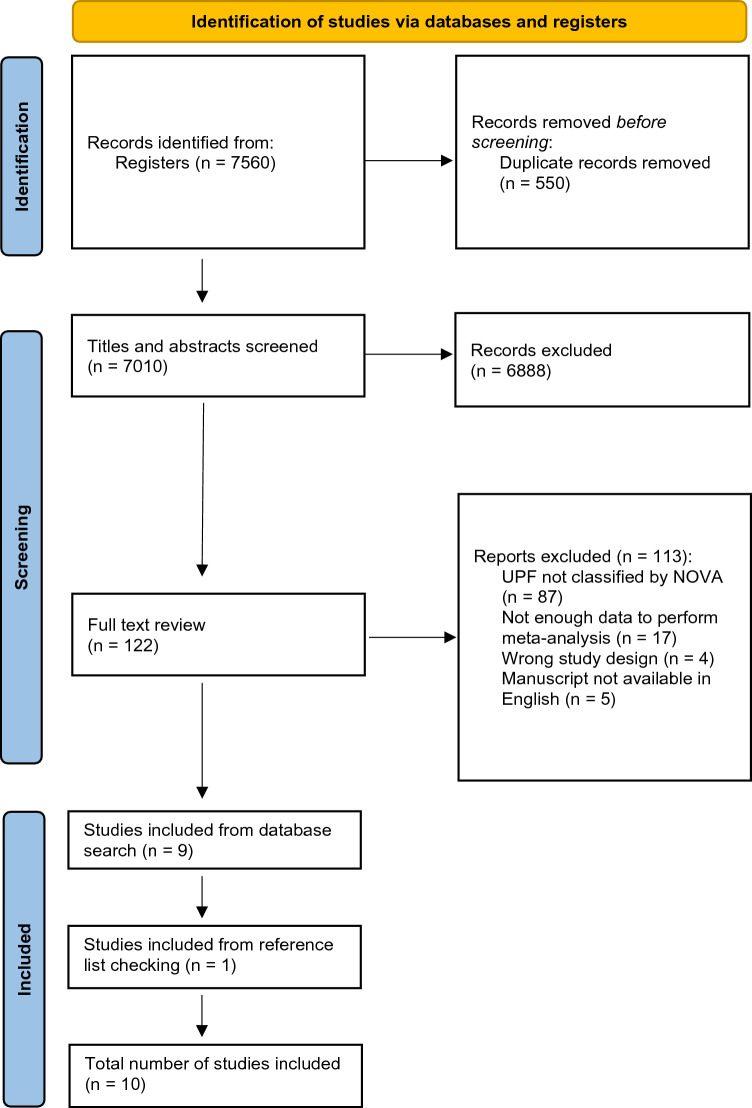
Preferred reporting items for systematic reviews and meta-analysis (PRISMA) reported flow diagram for study selection process

A flowchart demonstrating the selection process of the studies is illustrated in Fig. 1. After duplicates were excluded (n = 550), 7010 records were identified and nine met inclusion criteria. A further single study was included after searching reference lists, bringing the total number of included studies to ten. In total, 867,316 participants were analysed. In full text review, most studies were excluded due to not allowing classification of UPFs via NOVA (n = 87) or lacking sufficient data to perform meta-analysis (n = 17).

The main demographics and results of the included studies are shown in Table 1. The studies included participants from Asia [30, 31], USA [32–34] and Europe [21, 35–38]. Eight studies were longitudinal, with follow-up ranging from 6.8 to 22 years [33, 37], one was case–control [36], and one was cross-sectional in design [31]. Sample sizes ranged from 108 to 493,888 [36, 38]. All studies adjusted for age and sex, alongside other confounders such as SES (including education level) (n = 9), TEI (n = 7), body mass index (BMI) (n = 9), CVD (n = 9) and T2D (n = 9). Nine studies included both males and females, with one study including only males [37]. One study directly referenced NOVA [21], with nine studies allowing indirect assessment of foods named as ultra-processed by NOVA. This included dietary patterns that were rich in UPFs [31–33], and focus on specific UPFs such as processed meats [35, 37, 38] or SSBs [34]. Three studies used data from the UK biobank, with two commenting on processed meats [35, 38] and one on all UPFs [21]. All studies collected dietary information via Food Frequency Questionnaires (FFQs). Seven studies evaluated the association between UPFs and dementia [21, 32, 34–38], and three with MCI [30, 31, 33]. Dementia was classified according to International Classification of Diseases (ICD) in five articles [21, 32, 35, 37, 38], with the Diagnostic and Statistical Manuscript of Mental Disorders (DSM) being used for two studies [34, 36]. For MCI, validated screening items were used [30, 31, 33]. All studies were published after 2017.

**Table 1:** Description of studies included in the systematic review (*n* = 10)

Publication	Source of data and study design (follow up)	Population (age/sex)	Dementia diagnosis	Exposure (via NOVA unless otherwise stated)	Adjustment	Outcome
Dearborn-Tomazos et al. 2019 [32]	Atherosclerosis Risk in Communities (ARIC) Prospective cohort (20 years)	15,792 males/females, mean age 54.6 years	ICD-9 and ICD-10 criteria for dementia	Western dietary pattern: Moderate: 2° tertile High: 3° tertile	Age, sex, education, race-field centre, total calories, apolipoprotein E e4 status, alcohol use, smoking, activity level, BMI, total cholesterol, coronary heart disease, hypertension, diabetes, stroke	Dementia: Moderate 1.01 (0.91–1.13) and high intake 1.06 (0.92–1.22) had no significant association
Dobreva et al. 2022 [35]	UK Biobank Retrospective cohort (11.4 years)	249,511 males/females, mean age 62	ICD-9 and ICD-10 criteria for dementia	Processed meats: Moderate: 3° quintile High: 5° quintile	Age, sex, Townsend deprivation score, age left education, household income, physical activity, smoking status, weekly alcohol units, loneliness, depression, BMI, cholesterol, diabetes, hypertension, cardiovascular events, major dietary changes	Dementia: Moderate 0.83 (0.72–0.95) and high intake 1.07 (0.88–1.30) had no significant association
Feng et al. 2020 [30]	China Health and Nutrition Survey Retrospective cohort (18 years)	8236 males/females, mean age 62.98 years	Cognitive Screening Item: Telephone Interview for Cognitive Status (< 7)	Fast-food, SSBs, salty snacks: Moderate: No data High: High intake	Age, sex, smoking, drinking, ethnicity, education levels, married status, regions, gross family income, hypertension, diabetes, death, BMI and physical activity	MCI: High intake increased the risk from 28% to over twofold Fast-foods 2.40 (1.76–3.28) SSBs 1.28 (1.02–1.61) Salty-snacks 1.52 (1.17–1.96)
Filippini et al. 2020 [36]	Newly-diagnosed patients referred to the Cognitive Neurology Network of Modena province Case–control	108 males/females, mean age 65 years	DSM-V criteria for dementia	Pizza, salty snacks, processed meats, sweets, chocolates, cakes, pastries, ice cream, SSBs: Moderate: 2° tertile High: 3° tertile	Sex, age, educational attainment, and energy intake	Dementia: Moderate and high intake had no significant association Moderate intake: Pizza and salty snacks 0.33 (0.11–0.84) Processed meats 0.51 (0.18–1.47) Sweets, chocolates, cakes etc. 1.47 (0.50–4.38) SSBs 1.67 (0.38–7.40) High intake: Pizza and salty snacks 0.33 (0.11–0.84) Processed meats 0.94 (0.35–2.54) Sweets, chocolates, cakes etc. 2.61 (0.82–8.34) SSBs 0.62 (0.18–2.18)
Fu et al. 2022 [31]	Tianjin Elderly Nutrition and Cognition Cohort (TENCC) Cross-sectional	4457 males/females, mean age 67.6 years	Petersen criteria (5 out of 5 of the criteria needed)	Processed foods dietary pattern: Moderate: 2° quartile High: 4° quartile	Sex, age, educational level, income, marital status, BMI, PA, hypertension, diabetes, hyperlipidemia, GS, smoking status, drinking status, and total energy intake	MCI: Moderate intake 1.32 (0.97–1.79) had no significant association High intake 1.39 (1.03–1.88) increased by 39%
Li et al. 2019	UK Biobank Prospective cohort (10 years)	72,083 males/females, mean age	ICD-10 criteria for dementia	Moderate: 2° quartile High: 4° quartile	Age, sex, education level, total daily intake, smoking, alcohol intake, physical activity, BMI, sleep duration, cardiovascular disease, family history of dementia, healthy diet score	Dementia: Moderate intake 1.02 (0.80–1.31) had no significant association High intake 1.44 (1.12–1.85) increased the risk by 44%
Pearson et al. 2016 [33]	REGARDS Prospective cohort (6.8 years)	18,080 males/females, mean age 64.8 years	Six-item Screener (SIS) (shifting from intact cognitive function to impaired cognitive function (a score ≤ 4))	Southern dietary pattern: Moderate: 3° quintile High: 5° quintile	Age, race, sex, region, total energy intake, income, education, physical activity, smoking status, BMI, hypertension, diabetes, cardiovascular disease and depressive symptoms	MCI: Moderate 1.07 (0.81–1.33) and high intake 1.16 (0.93–1.45) had no significant association
Ylilauri et al. 2022 [37]	Kuopio Ischaemic Heart Disease Risk Factor Study (KIHD) Prospective cohort (22 years)	2497 males, aged 42–60 years	ICD-8, ICD-9 and ICD-10 criteria for dementia	Processed red meats: Moderate: 2° quartile High: 4° quartile	Age, baseline examination year, energy intake, education years, pack-years of smoking, BMI, diabetes, leisure-time physical activity, coronary heart disease, use of lipid-lowering medication, intakes of alcohol, fibre, sum of fruits, berries and vegetables, and dietary fat quality	Dementia: Moderate 1.06 (0.79–1.44) and high intake 1.12 (0.79–1.57) had no significant association
Zhang et al. 2021 [38]	UK Biobank Retrospective cohort (8 years)	493,888 males/females, mean age 56.5 years	ICD-9 and ICD-10 criteria for dementia	Processed meats: Moderate: 2° quartile High: 4° quartile	Age, gender, ethnicity, socioeconomic status, educational level, region, BMI, physical activity, smoking status, typical sleep duration, stroke history, family history of dementia, and dietary factors including total consumption of vegetables and fruits, total fish, tea and coffee, and alcohol	Dementia: Moderate intake 1.13 (1.02–1.25) increased the risk by 13% High intake 1.67 (1.41–1.98) increased the risk by 67%
Miao et al. 2021 [34]	Framingham Heart Study (FHS) Retrospective cohort (19 years)	2664 males/females, mean age 53.7 years	DSM-IV criteria for dementia	SSBs: Moderate: 2° tertile High: 3° tertile	Age, sex, hypertension, smoking, BMI, diabetes	Dementia: Moderate intake 1.53 (1.21–1.93) increased the risk by 53% High intake 2.80 (2.24–3.50) increased the risk over twofold

The outcome column represents the odds ratio or relative risk calculated by the authors of the original included studies

*ICD* International Classification of Diseases, *DSM* Diagnostic and Statistical Manuscript of Mental Disorders

Supplementary Table S4 shows the quality of evidence as reported by the NOS. The studies had NOS scores between 6 and 8, with a mean of 7.5. Nine studies were considered high quality, with one study considered moderate quality and higher risk of bias [30]. Supplementary Table S5 shows the NutriGrade evaluation of evidence credibility which is considered high with an overall score of 9.

### Systematic review

Three out of seven studies reported significant associations between high UPF consumption and the development of dementia (excluding MCI) [21, 34, 38]. The four non-significant studies reported a trend towards significance for dementia risk [32, 35–37]. Two studies reported significant associations between high UPF consumption and the development of MCI [30, 31]. The one non-significant study reported a trend towards significance [33]. Two studies reported significant associations between UPF consumption and the development of all-cause dementia with moderate intake of UPFs [34, 38].

### Association between ultra-processed food intake and dementia

**Fig. 2:**
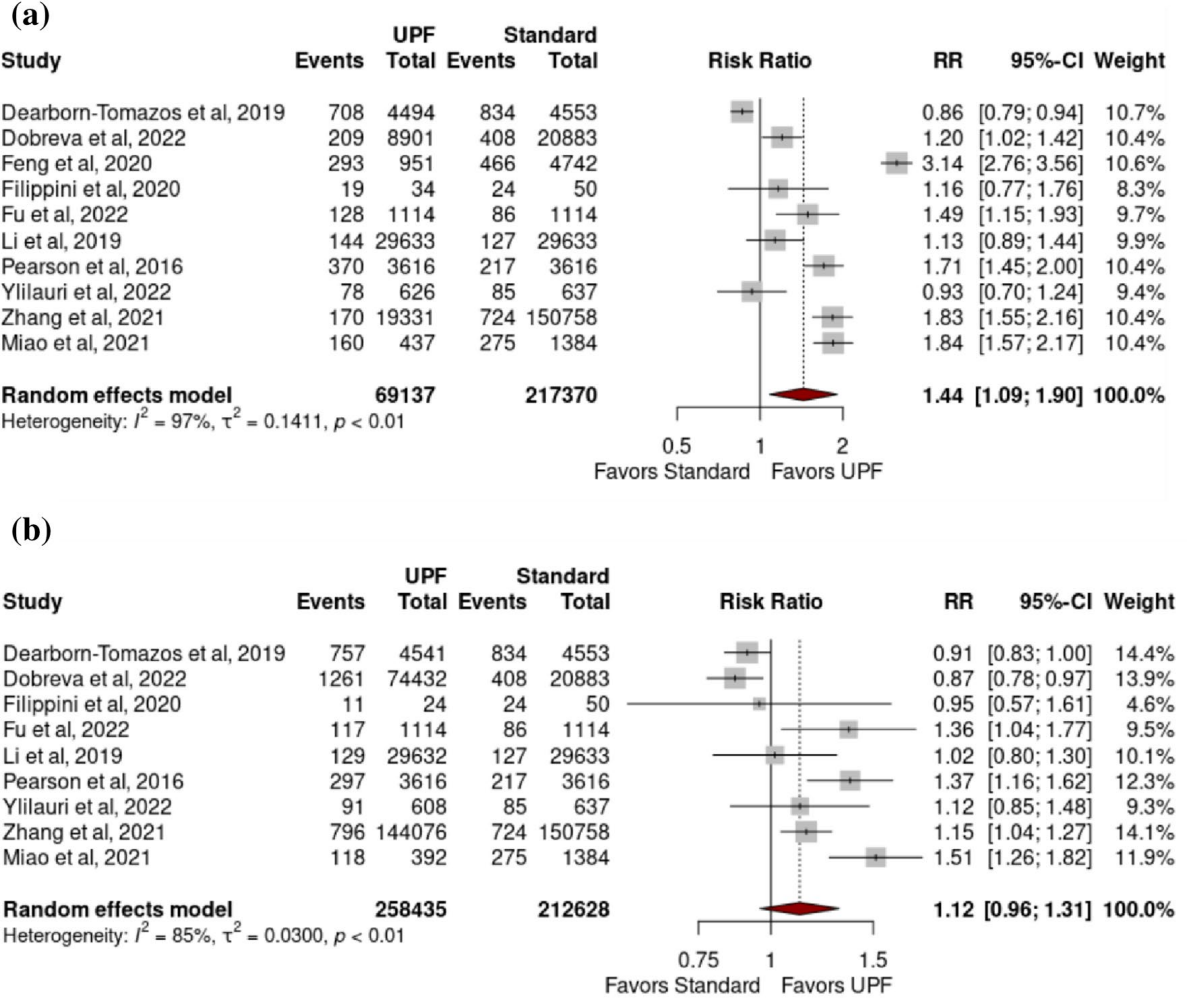
Forest plots from a random-effects model portraying the association between **a** high (vs low) ultra-processed food intake and development of all-cause dementia; **b** moderate ultra-processed food intake and development of all-cause dementia

*All cause dementia:* High intake of UPFs was associated with increased risk of developing all-cause dementia (pooled RR 1.44 (1.09–1.90) (p = 0.02)), with high heterogeneity (97.0% (p < 0.01)) (Fig. 2a). Moderate intake of UPFs was not associated with increased risk of developing all-cause dementia (pooled RR 1.12 (0.96–1.31) (p = 0.13)), with high heterogeneity (I^2^ = 85.0% (p =  < 0.01)) (Fig. 2b).

**Fig. 3:**
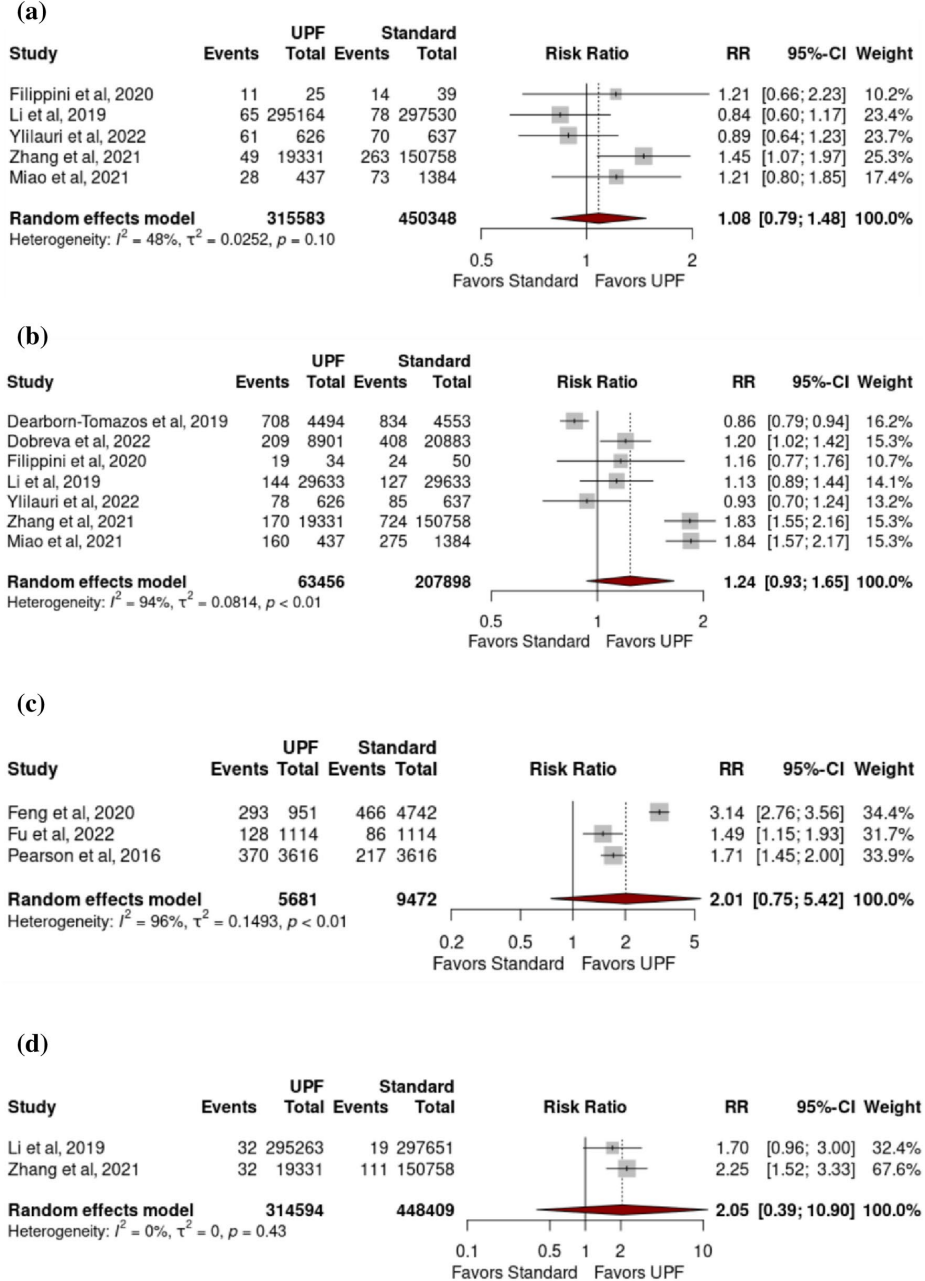
Forest plots from a random-effects model portraying the association between **a** high ultra-processed food intake and development of dementia (excluding mild cognitive impairment); **b** high ultra-processed food intake and development of mild cognitive impairment; **c** high ultra-processed food intake and development of Alzheimer dementia; **d** high ultra-processed food intake and development of vascular dementia

*Dementia sub-types:* No significant associations existed between high intake of UPFs and any of the following: dementia excluding MCI (pooled RR 1.24 (0.93–1.65) (p = 0.11)) (I^2^ = 94.2% (p =  < 0.01)), AD (1.08 (0.79–1.48) (0.52) (48.3% (0.10)), VD (2.05 (0.39–10.90) (0.12) (0.0% (0.43)) or MCI (2.01 (0.75–5.42) (0.09)) (95.7% (< 0.01)) (Fig. 3a–d).

Bubble plots demonstrate the dose–response relationship between UPF intake and all-cause dementia (Supplementary Fig. S5).

### Sensitivity analysis

Significant associations remained between high UPF intake and all-cause dementia when: NOVA was not directly referenced (1.47 (1.08–2.01) (0.02)) (97.3% (< 0.01)), longitudinally designed (1.47 (1.02–2.11) (0.04)) (97.7% (< 0.01)) and low risk of bias (1.31 (1.05–1.64) (0.02)) (93.7% (< 0.01)). When the study by Dobreva et al. was excluded owing to using a similar cohort from the UK Biobank as the larger study by Zhang et al., significant associations remained (1.46 (1.07–2.01) (0.02)) (97.3% (< 0.01))[35, 38]. All other sensitivity analyses produced non-significant results and are presented in Supplementary Table S6 with corresponding forest plots in Supplementary Fig. S1 and funnel plots in Supplementary Fig. S2.

### Adjusting for confounders

Significant associations remained between high UPF intake and all-cause dementia when studies adjusted for BMI (1.46 (1.07–2.00) (0.02)) (97.3% (< 0.01)), CVD (1.46 (1.07–2.00) (0.02)) (97.3% (< 0.01)) and SES (1.39 (1.02–1.90) (0.04)) (97.2% (< 0.01)). When studies adjusted for T2D (1.47 (0.97–2.00) (0.06)) (97.9% (< 0.01)) and TEI (1.26 (0.95–1.67) (0.09)) (93.9% (< 0.01)), significant associations were lost. Forest plots are presented in Supplementary Fig. S3 and funnel plots in Supplementary Fig. S4.

### Publication bias

**Fig. 4:**
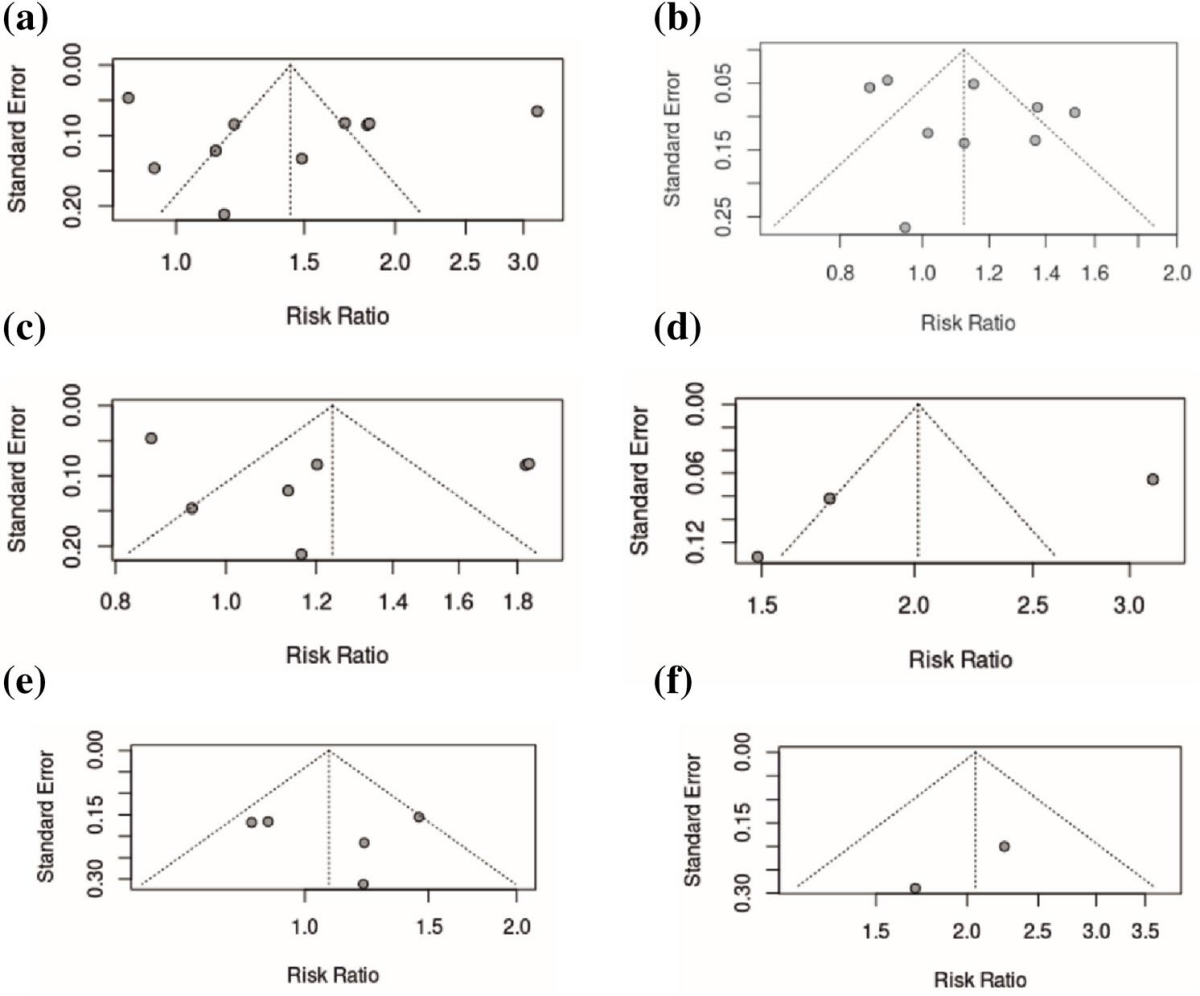
Funnel plots portraying risk of publication bias for studies assessing the association between **a** high ultra-processed food intake and development of all-cause dementia; **b** moderate ultra-processed food intake and development of all-cause dementia; **c** high ultra-processed food intake and development of dementia (excluding mild cognitive impairment); **d** high ultra-processed food intake and development of mild cognitive impairment; **e** high ultra-processed food intake and development of Alzheimer dementia; **f** high ultra-processed food intake and development of vascular dementia

Begg’s funnel plots visually represent publication bias among the included studies. Funnel plots suggest low risk of publication bias with good relative symmetry (Fig. 4).

## Discussion

We present the first systematic review and meta-analysis to assess the association between ultra-processed food consumption and dementia, convincingly demonstrating that high UPF intake is associated with dementia and suggesting that ultra-processed diets could contribute to cognitive impairment. However, there was not a statistically significant relationship between moderate intake of UPFs and dementia, meaning we did not demonstrate a robust dose–response relationship between the quantity of UPFs consumed and dementia prevalence.

The beneficial effect of diet quality on cognitive health has previously been explored [14, 15]. The relationship between UPFs and dementia is likely multifactorial and related to several inter-related mechanisms including indirectly from the development of cardiovascular risk factors such as hypertension/hyperlipidaemia [42–44], obesity and T2D [46] and directly from the consumption of high energy–density foods with inherent poor macro- and micronutrient profiles [40] and harmful chemical additives that together can alter gut microbial diversity [47]. Macronutrients relevant include dietary saturated fat, trans-fat, refined carbohydrates and low dietary fibre [45]. Micronutrients relevant include high sodium salt content in UPFs [48, 49]. Finally, artificial additives to food [10], such as monosodium glutamate [51], and packaging, such as bisphenol A [52], may accelerate cognitive decline, highlighted by artificially sweetened beverages demonstrating associations with all-cause dementia [53]. Possible biological pathways include pro-inflammatory adipokine and hormone secretion that promote neurodegeneration through amyloid deposition, vascular remodelling [39], cerebral microvascular dysfunction [50] and brain insulin resistance [40, 41].

The association of UPFs with other markers of poor metabolic health has been shown for a variety of conditions supporting the biological plausibility of our findings. A strong association exists between UPFs and multiple cardiometabolic diseases such as obesity, T2D, CVD, metabolic-associated steatotic liver disease and at least thirteen hormone dependent cancers [16–20]. The impact of this UPF consumption on health and disease is frequently characterised by end-organ damage including the liver, heart and kidneys. The brain appears also to be a target for such end-organ damage. Included studies adjusted for a similar battery of confounding cardiometabolic variables and significant results remained when sensitivity analyses were performed using studies adjusted for BMI, CVD and SES (Supplementary Fig. S3). Of course, RCTs assessing the association between UPFs and incident cognitive impairment/dementia would be ethically unjustifiable considering the associations described above between UPFs and cardiometabolic disease [16, 18, 19]. Hence the current review synthesises best available data to provide this evidence.

Although we provide strong evidence for a high intake of UPFs being associated with dementia, between-study heterogeneity was observed in respect to classification of UPF, study design, sample size, method of diagnosing dementia and participant age. To this end, we performed sensitivity analyses to identify the impact of heterogeneity on our results (Supplementary Table S6). Classifying UPFs in the original studies lacked standardisation. One study referenced NOVA in its methodology, whilst nine assessed various foods that were retrospectively defined as ultra-processed by our research team using NOVA criteria. Ultra-processing of specific foods (i.e., processed meats [38]) may increase dementia risk more than others. Similarly, this meant there was no pre-determined cut-off to quantify exposure. Moderate intake in one population may exceed high intake in another. Despite this, significant associations remained between high intake of UPF and dementia when sensitivity analysis was performed in respect to UPF classification (Supplementary Fig. S1a). Funnel plots highlight greater effect size range in studies with small sample sizes, and results lose significance when performing sensitivity analysis based on a sample size cut-off point of 10,000 participants (Supplementary Fig. S1d & e). In addition, studies with AD/VD as the outcome used ICD or DSM codes, whereas no studies with MCI as the outcome did so despite codes existing, and all included studies assessed diet using FFQs which increases recall bias and underreporting of true intake [54]. Together, this highlights the need for more robust population-based research assessing the association between UPF and dementia using a standardised classification approach.

What are the implications of our findings? To optimise brain health with aging, we need to consider strategies to reduce global UPF consumption. Mexico, UK and South Africa have all implemented sugar-based taxes on SSBs and packaged foods [55, 56] based on the premise that taxation of UPFs could subsidise the cost of minimally processed foods. Careful political consideration would be needed to spread the distribution of fresh produce equally so as not to disproportionately favour economically advanced nations.

In conclusion, the current systematic review and meta-analysis highlighted an association between the consumption of a high intake of UPFs and dementia. Further robust population-level characterisation of UPF consumption using the standardised NOVA classification system is required to determine more precisely the magnitude of, and the temporal relationship between, UPF intake and dementia. However, our findings highlight the contributory role of UPF consumption to the development of dementia and that co-ordinated global and national public health policies and clinical guidelines are needed to displace consumption of UPFs with fresh, minimally processed, easily affordable food, to tackle the societal burden of dementia.

### Supplementary Information

Below is the link to the electronic supplementary material.

Supplementary file1 (DOCX 27578 KB)

## Data Availability

Data, analytical methods and study materials can be made available to other researchers if requested.

## References

[1] Diagnostic criteria for dementia. 2023 29.08.2023. https://www.dementia.org.au/information/for-health-professionals/clinical-resources/diagnostic-criteria-for-dementia

[2] Jiang T (2013). Epidemiology and etiology of Alzheimer’s disease: from genetic to non-genetic factors. Curr Alzheimer Res.

[3] WHO (2021) Dementia. https://www.who.int/news-room/fact-sheets/detail/dementia. Cited 18 Dec 2022.

[4] Livingston G (2017). Dementia prevention, intervention, and care. Lancet.

[5] Machado-Fragua MD (2022). Association of metabolic syndrome with incident dementia: role of number and age at measurement of components in a 28-year follow-up of the Whitehall II cohort study. Diabetes Care.

[6] Hao Z (2022). Association between socioeconomic status and prevalence of cardio-metabolic risk factors: a cross-sectional study on residents in North China. Fronti Cardiovasc Med.

[7] Fischer C (2009). Impact of socioeconomic status on the prevalence of dementia in an inner city memory disorders clinic. Int Psychogeriatr.

[8] Jitlal M (2021). The influence of socioeconomic deprivation on dementia mortality, age at death, and quality of diagnosis: a nationwide death records study in England and Wales 2001–2017. J Alzheimers Dis.

[9] Monteiro CA (2013). Ultra-processed products are becoming dominant in the global food system. Obes Rev.

[10] Monteiro CA (2019). Ultra-processed foods: what they are and how to identify them. Public Health Nutr.

[11] Baker P (2020). Ultra-processed foods and the nutrition transition: global, regional and national trends, food systems transformations and political economy drivers. Obes Rev.

[12] Newton S, Braithwaite D, Akinyemiju TF (2017). Socio-economic status over the life course and obesity: systematic review and meta-analysis. PLoS ONE.

[13] Moubarac JC (2014). Food classification systems based on food processing: significance and implications for policies and actions: a systematic literature review and assessment. Curr Obes Rep.

[14] Fu J (2022). Association between the mediterranean diet and cognitive health among healthy adults: a systematic review and meta-analysis. Front Nutr.

[15] Cao L (2016). Dietary patterns and risk of dementia: a systematic review and meta-analysis of cohort studies. Mol Neurobiol.

[16] Delpino FM (2022). Ultra-processed food and risk of type 2 diabetes: a systematic review and meta-analysis of longitudinal studies. Int J Epidemiol.

[17] Chen X (2020). Consumption of ultra-processed foods and health outcomes: a systematic review of epidemiological studies. Nutr J.

[18] Lane MM (2021). Ultraprocessed food and chronic noncommunicable diseases: a systematic review and meta-analysis of 43 observational studies. Obes Rev.

[19] Askari M (2020). Ultra-processed food and the risk of overweight and obesity: a systematic review and meta-analysis of observational studies. Int J Obes (Lond).

[20] Henney AE (2023). Ultra-processed food intake is associated with non-alcoholic fatty liver disease in adults: a systematic review and meta-analysis. Nutrients.

[21] Li H (2022). Association of ultraprocessed food consumption with risk of dementia: a prospective cohort study. Neurology.

[22] Cardoso BR, Machado P, Steele EM (2022). Association between ultra-processed food consumption and cognitive performance in US older adults: a cross-sectional analysis of the NHANES 2011–2014. Eur J Nutr.

[23] Weinstein G (2022). Consumption of ultra-processed food and cognitive decline among older adults with type-2 diabetes. J Gerontol A Biol Sci Med Sci.

[24] Hutton B (2015). The PRISMA extension statement for reporting of systematic reviews incorporating network meta-analyses of health care interventions: checklist and explanations. Ann Intern Med.

[25] Stang A (2010). Critical evaluation of the Newcastle–Ottawa Scale for the assessment of the quality of nonrandomized studies in meta-analyses. Eur J Epidemiol.

[26] Schwingshackl L (2016). Perspective: NutriGrade: a scoring system to assess and judge the meta-evidence of randomized controlled trials and cohort studies in nutrition research. Adv Nutr.

[27] Lachin JM (2013). Power of the Mantel–Haenszel and other tests for discrete or grouped time-to-event data under a chained binomial model. Stat Med.

[28] Higgins JP, Thompson SG (2002). Quantifying heterogeneity in a meta-analysis. Stat Med.

[29] Jackson D (2017). Paule–Mandel estimators for network meta-analysis with random inconsistency effects. Res Synth Methods.

[30] Feng T (2020). Associations of health behaviors, food preferences, and obesity patterns with the incidence of mild cognitive impairment in the middle-aged and elderly population: an 18-year cohort study. J Affect Disord.

[31] Fu J (2022). Association between methionine cycle metabolite-related diets and mild cognitive impairment in older Chinese adults: a population-based observational study. Nutr Neurosci.

[32] Dearborn-Tomazos JL (2019). Association of dietary patterns in midlife and cognitive function in later life in US adults without dementia. JAMA Netw Open.

[33] Pearson KE (2016). Dietary patterns are associated with cognitive function in the REasons for Geographic And Racial Differences in Stroke (REGARDS) cohort. J Nutr Sci.

[34] Miao H (2021). Sugar in beverage and the risk of incident dementia, Alzheimer’s disease and stroke: a prospective cohort study. J Prev Alzheimers Dis.

[35] Dobreva I, Marston L, Mukadam N (2022). Which components of the Mediterranean diet are associated with dementia? A UK Biobank cohort study. GeroScience.

[36] Filippini T (2020). Dietary habits and risk of early-onset dementia in an Italian case-control study. Nutrients.

[37] Ylilauri MPT (2022). Associations of dairy, meat, and fish intakes with risk of incident dementia and with cognitive performance: the Kuopio Ischaemic Heart Disease Risk Factor Study (KIHD). Eur J Nutr.

[38] Zhang H (2021). Meat consumption and risk of incident dementia: cohort study of 493,888 UK Biobank participants. Am J Clin Nutr.

[39] Qu Y (2020). Association of body mass index with risk of cognitive impairment and dementia: a systematic review and meta-analysis of prospective studies. Neurosci Biobehav Rev.

[40] Roberts RO (2012). Relative intake of macronutrients impacts risk of mild cognitive impairment or dementia. J Alzheimers Dis.

[41] Kellar D, Craft S (2020). Brain insulin resistance in Alzheimer’s disease and related disorders: mechanisms and therapeutic approaches. Lancet Neurol.

[42] Ruan Y (2018). Dietary fat intake and risk of Alzheimer’s disease and dementia: a meta-analysis of cohort studies. Curr Alzheimer Res.

[43] Barnard ND, Bunner AE, Agarwal U (2014). Saturated and trans fats and dementia: a systematic review. Neurobiol Aging.

[44] Cao GY (2019). Dietary fat intake and cognitive function among older populations: a systematic review and meta-analysis. J Prev Alzheimers Dis.

[45] Yamagishi K (2022). Dietary fiber intake and risk of incident disabling dementia: the circulatory risk in communities study. Nutr Neurosci.

[46] Reynolds A (2019). Carbohydrate quality and human health: a series of systematic reviews and meta-analyses. Lancet.

[47] Saji N (2019). Proportional changes in the gut microbiome: a risk factor for cardiovascular disease and dementia?. Hypertens Res.

[48] Martini D (2021). Ultra-processed foods and nutritional dietary profile: a meta-analysis of nationally representative samples. Nutrients.

[49] Mohan D (2020). Link between dietary sodium intake, cognitive function, and dementia risk in middle-aged and older adults: a systematic review. J Alzheimers Dis.

[50] Boegehold MA (2013). The effect of high salt intake on endothelial function: reduced vascular nitric oxide in the absence of hypertension. J Vasc Res.

[51] Fuchsberger T (2019). Oral monosodium glutamate administration causes early onset of Alzheimer’s disease-like pathophysiology in APP/PS1 mice. J Alzheimers Dis.

[52] Wang T (2017). Involvement of insulin signaling disturbances in bisphenol A-induced Alzheimer’s disease-like neurotoxicity. Sci Rep.

[53] Pase MP (2017). Sugar- and artificially sweetened beverages and the risks of incident stroke and dementia: a prospective cohort study. Stroke.

[54] Pérez Rodrigo C (2015). Food frequency questionnaires. Nutr Hosp.

[55] Colchero MA (2016). Beverage purchases from stores in Mexico under the excise tax on sugar sweetened beverages: observational study. BMJ.

[56] Colchero MA (2017). In Mexico, evidence of sustained consumer response two years after implementing a sugar-sweetened beverage tax. Health Aff (Millwood).

