# Supplementary material for: High intake of ultra-processed food is associated with dementia in adults: a systematic review and meta-analysis of observational studies
Source: J Neurol. 2023 Oct 13;271(1):198–210. doi: 10.1007/s00415-023-12033-1 (PMC10770002; doi:10.1007/s00415-023-12033-1)
Supplement: Supplementary file 1 — Supplementary file1 (DOCX 27578 KB) [file 415_2023_12033_MOESM1_ESM.docx]

**Supplementary table S1**: NOVA classification of ultra-processed foods

**Supplementary Table S2**: Table of search terms used during the systematic searching of databases.

**Supplementary Table S3**: Inclusion and exclusion criteria used in the protocol for the selection of papers from the systematic search. UPF = ultra-processed food; MCI = mild cognitive impairment; AD = Alzheimer disease; VD = vascular dementia; OR = odds ratio; RR = relative risk; HR = hazard ratio.

**Supplementary table S4:** **a)** Quality of evidence of longitudinally designed studies as reported by Newcastle Ottawa Scale (NOS) (n=2), **b)** quality of evidence of case-control studies as reported by NOS (n=3), **c)** quality of evidence of cross-sectionally designed studies as reported by an adapted version of NOS (n=1).

| **ID** | **Representativeness of exposed cohort** | **Selection of non-exposed cohort** | **Ascertainment of exposure** | **Outcome of interest not present at start of study** | **Comparability of cohorts** | **Assessment**  **of outcome** | **Follow up length** | **Follow up rate** | **Score** |
| --- | --- | --- | --- | --- | --- | --- | --- | --- | --- |
| Dearborn-Tomazos et al | a | a | c | - a | a | b | a | b | 8 |
| Dobreva et al | a | a | c | a | a | b | a | b | 8 |
| Feng et al | a | a | c | a | a | c | a | c | 6 |
| Li et al | a | a | c | a | a | b | a | b | 8 |
| Pearson et al | a | a | c | a | a | c | a | b | 7 |
| Ylilauri et al | b | a | c | a | a | b | a | b | 8 |
| Zhang et al | a | a | c | a | a | b | a | b | 8 |
| Miao et al | a | a | c | a | a | b | a | b | 8 |

***a)***

***b)***

| **ID** | **Adequacy of case definition** | **Representativeness of cases** | **Selection of controls** | **Definition of controls** | **Comparability of cases and controls** | **Ascertainment of exposure** | **Same method for cases and controls** | **Response rate** | **Score** |
| --- | --- | --- | --- | --- | --- | --- | --- | --- | --- |
| Filippini et al | a | a | b | a | a | d | a | b | 6 |

***c)***

| **ID** | **Representativeness of sample** | **Sample size** | **Response rate** | **Ascertainment of exposure** | **Comparability of subjects in different outcome groups** | **Ascertainment of outcome** | **Statistics** | **Score** |
| --- | --- | --- | --- | --- | --- | --- | --- | --- |
| Fu et al [1] | a | a | b | b | a | b | a | 8 |

| **Item** | **Score** |
| --- | --- |
| **1** | 2 |
| **2** | 1 |
| **3** | 1 |
| **4** | 1 |
| **5** | 1 |
| **6** | 1 |
| **7** | 1 |
| **8** | 1 |
| **Total score** | 9 |
| **Credibility** | High |

**Supplementary table S5:** Credibility of evidence as reported by the NutriGrade tool (n=7). Item 1 risk of bias, study quality and limitations (0-2 points) - Quality of evidence as per Newcastle Ottawa Scale (mean) 7 or above = 2 points; Item 2 precision (0-1 point) - ≥500 events and the 95% CI excludes the null value; or ≥500 events, but 95% CI overlaps the null value, and 95% CI excludes important harm (RR <1.2) = 1 point; Item 3 heterogeneity (0-1 point) - 2 to 5 studies = 0 points, ≥10 studies, heterogeneity measures adequately reported, random-effects models, and subgroups analyses were conducted = 1 point; Item 4 directness (0-1 point) - no important differences in the population or intervention; hard clinical outcome = 1 point; Item 5 publication bias (0 to 1 point) - <5 studies = 0 points; no evidence for publication bias with test or plot (≥10 studies) = 1 point; Item 6 funding bias (0 to 1 point) - report from academic or research institution = 1 point; Item 7 effect size (0 to 2 points) - RR >1.20 and corresponding test statistically significant (highest vs lowest category) = 1 point; Item 8 dose-response (0 to 1 point) - no dose-response analysis = 0 point; significant linear dose-response relation = 1 point.

| **Sensitivity analysis** | **Pooled effect size (RR (95% ci) (p))** | **Heterogeneity (i^2^ = 95% (p))** |
| --- | --- | --- |
| Not reported as NOVA directly | 1.47 (1.08-2.01) (0.02) | 97.3% (<0.01) |
| **Study design** | | |
| Longitudinally designed | 1.47 (1.02-2.11) (0.04) | 97.3% (<0.01) |
| Non-longitudinally designed | 1.39 (0.34-5.66) (0.21) | 0.0% (0.33) |
| **Sample size** | | |
| >1000 | 1.46 (1.07-2.00) (0.02) | 97.3% (<0.01) |
| >10,000 | 1.29 (0.86-1.91) (0.14) | 95.7% (<0.01) |
| <10,000 | 1.59 (0.89-2.86) (0.09) | 95.3 % (<0.01) |
| **Continent** | | |
| North America | 1.39 (0.49-3.95) (0.31) | 97.9% (<0.01) |
| Europe | 1.24 (0.90-1.71) (0.13) | 83.1% (<0.01) |
| Asia | 2.18 (0.02-247.36) (0.28) | 96.0% (<0.01) |
| **Quality of evidence** | | |
| High quality evidence | 1.31 (1.05-1.64) (0.02) | 93.7% (<0.01) |
| **UK Biobank co-linearity** | | |
| Excludes Dobreva et al | 1.46 (1.07-2.01) (0.02) | 97.3% (<0.01) |

**Supplementary Table S6**: Table summarising the findings of sensitivity analysis.

**Supplementary figure S1**: Forest plots following sensitivity analyses assessing the association between high ultra-processed food intake and risk of all-cause dementia when studies **a)** do not report UPFs via the NOVA classification system directly**, b)** are longitudinally designed, **c)** are not longitudinally designed studies, **d)** have sample sizes above 10,000, **e)** have sample sizes below 10,000, **f)** are conducted in America, **g)** are conducted in Europe, **h)** are conducted in Asia, **i)** have high quality evidence, **j)** exclude cohort report by Dobreva et al.


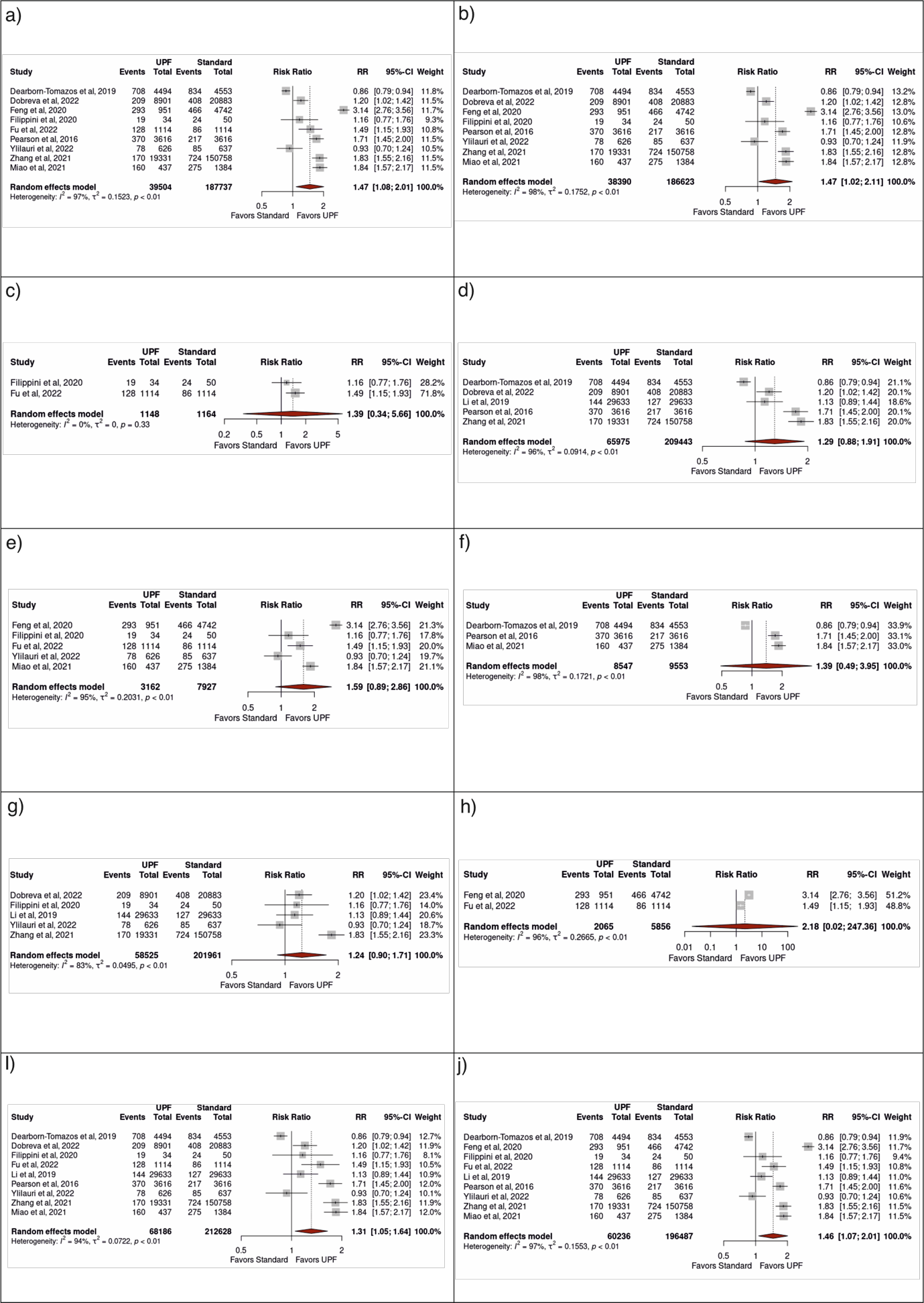


**Supplementary figure S2**: Funnel plots assessing the risk of bias in studies assessing the association between high ultra-processed food intake and risk of all-cause dementia when studies **a)** do not report UPFs via the NOVA classification system directly**, b)** are longitudinally designed, **c)** are not longitudinally designed studies, **d)** have sample sizes above 10,000, **e)** have sample sizes below 10,000, **f)** are conducted in America, **g)** are conducted in Europe, **h)** are conducted in Asia, **i)** have high quality evidence, **j)** exclude cohort report by Dobreva et al

**
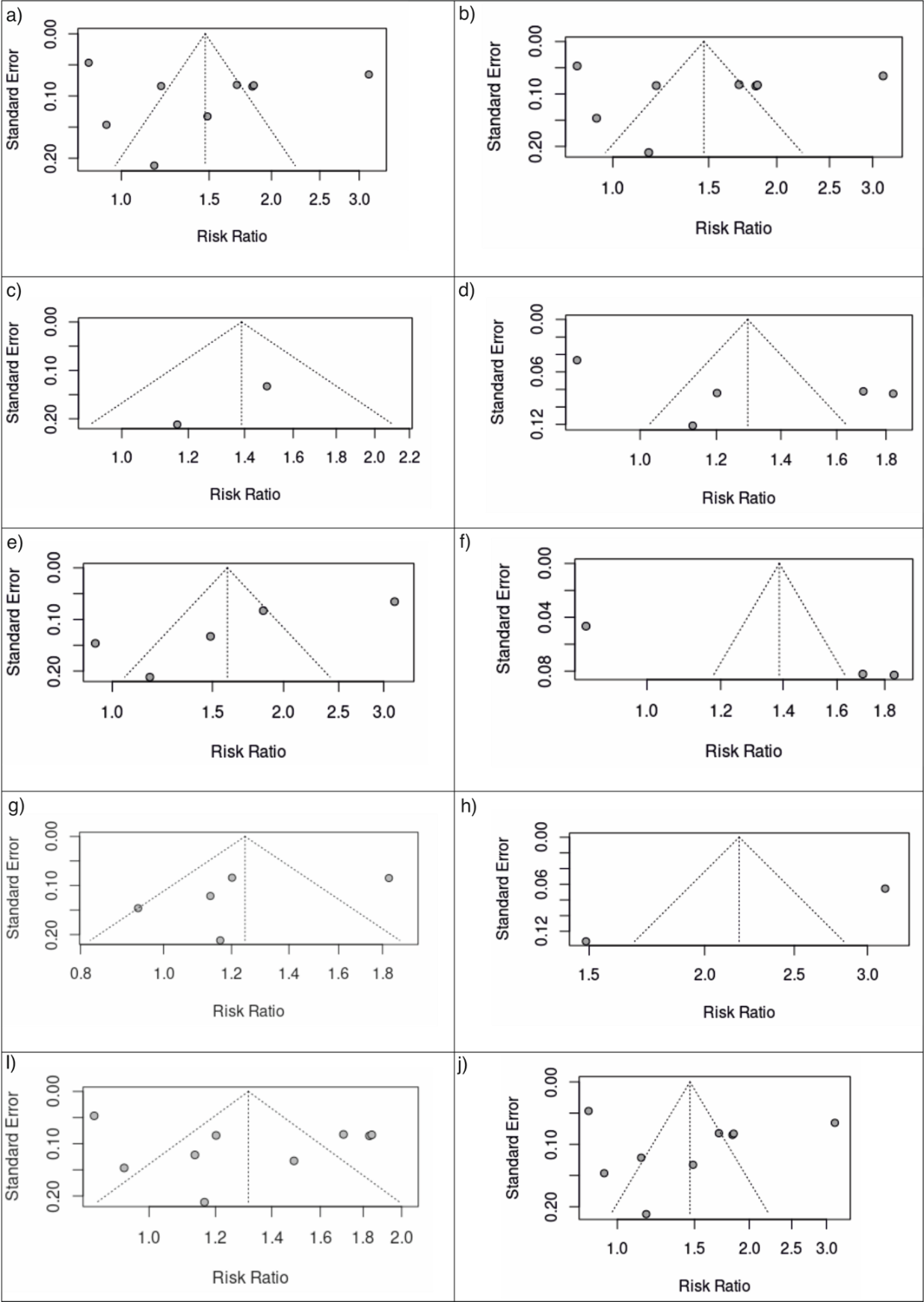
**

**Supplementary figure S3**: Forest plots assessing the association between high ultra-processed food intake and risk of all-cause dementia when studies adjust for **a)** body mass index (BMI)**, b)** total energy intake (TEI), **c)** socioeconomic status (SES), **d)** cardiovascular disease (CVD), **e)** type 2 diabetes (T2D)


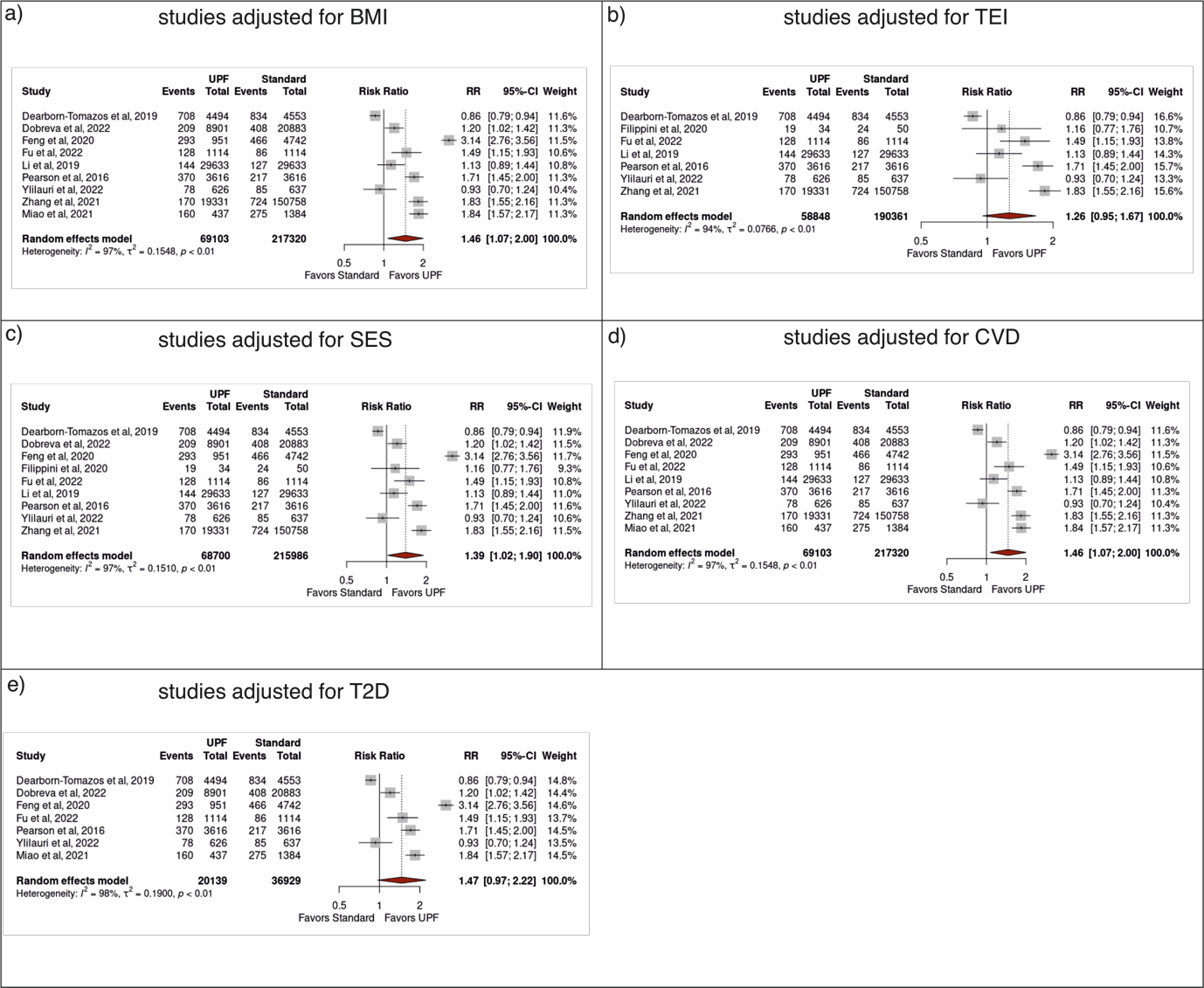


**Supplementary figure S4**: Funnel plots assessing the risk of bias in studies assessing the association between high ultra-processed food intake and risk of all-cause dementia when studies adjust for **a)** body mass index (BMI)**, b)** total energy intake (TEI), **c)** socioeconomic status (SES), **d)** cardiovascular disease (CVD), **e)** type 2 diabetes (T2D)


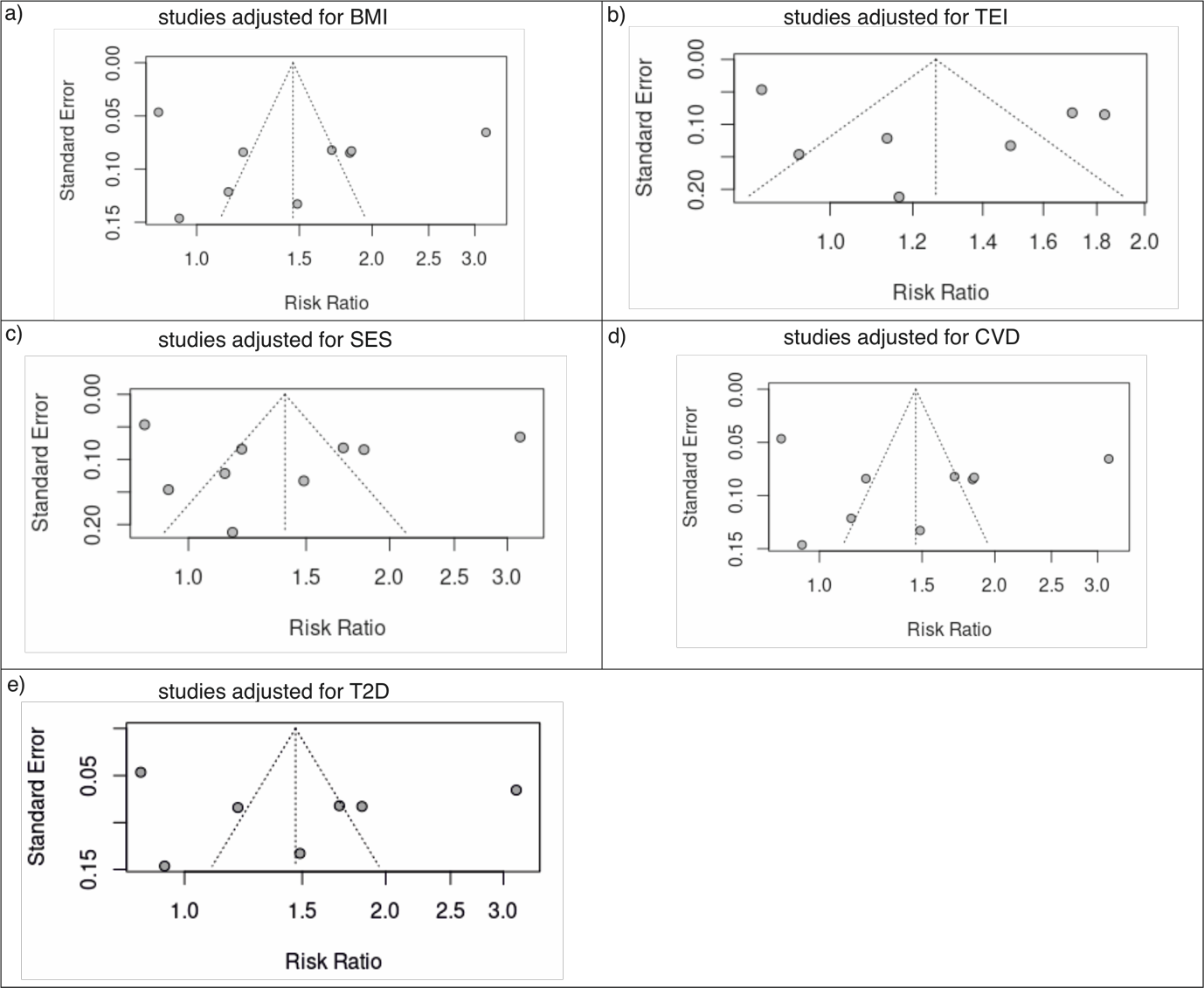


**Supplementary figure S5**: Bubble plots for meta-regression exploring the dose-response association between a) ultra-processed food intake and all-cause dementia; b) ultra-processed food intake and dementia (excluding mild cognitive impairment). The size of circle represents the sample size of individual included the studies.

| **Section and Topic** | **Item #** | **Checklist item** | **Reported (Yes/No)** |
| --- | --- | --- | --- |
| **TITLE** | | |  |
| Title | 1 | Identify the report as a systematic review. | Yes |
| **BACKGROUND** | | |  |
| Objectives | 2 | Provide an explicit statement of the main objective(s) or question(s) the review addresses. | Yes |
| **METHODS** | | |  |
| Eligibility criteria | 3 | Specify the inclusion and exclusion criteria for the review. | Yes |
| Information sources | 4 | Specify the information sources (e.g. databases, registers) used to identify studies and the date when each was last searched. | Yes |
| Risk of bias | 5 | Specify the methods used to assess risk of bias in the included studies. | Yes |
| Synthesis of results | 6 | Specify the methods used to present and synthesise results. | Yes |
| **RESULTS** | | |  |
| Included studies | 7 | Give the total number of included studies and participants and summarise relevant characteristics of studies. | Yes |
| Synthesis of results | 8 | Present results for main outcomes, preferably indicating the number of included studies and participants for each. If meta-analysis was done, report the summary estimate and confidence/credible interval. If comparing groups, indicate the direction of the effect (i.e. which group is favoured). | Yes |
| **DISCUSSION** | | |  |
| Limitations of evidence | 9 | Provide a brief summary of the limitations of the evidence included in the review (e.g. study risk of bias, inconsistency and imprecision). | Yes |
| Interpretation | 10 | Provide a general interpretation of the results and important implications. | Yes |
| **OTHER** | | |  |
| Funding | 11 | Specify the primary source of funding for the review. | NA |
| Registration | 12 | Provide the register name and registration number. | NA |

**Supplementary table S7:** PRISMA abstract checklist.

**Supplementary table S8:** PRISMA checklist

| **Section and Topic** | **Item #** | **Checklist item** | **Location where item is reported** |
| --- | --- | --- | --- |
| **TITLE** | | |  |
| Title | 1 | Identify the report as a systematic review. | Title page |
| **ABSTRACT** | | |  |
| Abstract | 2 | See the PRISMA 2020 for Abstracts checklist. | Page 2 |
| **INTRODUCTION** | | |  |
| Rationale | 3 | Describe the rationale for the review in the context of existing knowledge. | Page 4 |
| Objectives | 4 | Provide an explicit statement of the objective(s) or question(s) the review addresses. | Page 4 |
| **METHODS** | | |  |
| Eligibility criteria | 5 | Specify the inclusion and exclusion criteria for the review and how studies were grouped for the syntheses. | Page 5-6 |
| Information sources | 6 | Specify all databases, registers, websites, organisations, reference lists and other sources searched or consulted to identify studies. Specify the date when each source was last searched or consulted. | Page 5 |
| Search strategy | 7 | Present the full search strategies for all databases, registers and websites, including any filters and limits used. | Page 5 supplementary material (supplementary table S1) |
| Selection process | 8 | Specify the methods used to decide whether a study met the inclusion criteria of the review, including how many reviewers screened each record and each report retrieved, whether they worked independently, and if applicable, details of automation tools used in the process. | Page 6-7 |
| Data collection process | 9 | Specify the methods used to collect data from reports, including how many reviewers collected data from each report, whether they worked independently, any processes for obtaining or confirming data from study investigators, and if applicable, details of automation tools used in the process. | Page 7 |
| Data items | 10a | List and define all outcomes for which data were sought. Specify whether all results that were compatible with each outcome domain in each study were sought (e.g. for all measures, time points, analyses), and if not, the methods used to decide which results to collect. | Page 6-7 |
|  | 10b | List and define all other variables for which data were sought (e.g. participant and intervention characteristics, funding sources). Describe any assumptions made about any missing or unclear information. | Page 7 |
| Study risk of bias assessment | 11 | Specify the methods used to assess risk of bias in the included studies, including details of the tool(s) used, how many reviewers assessed each study and whether they worked independently, and if applicable, details of automation tools used in the process. | Page 7-8 |
| Effect measures | 12 | Specify for each outcome the effect measure(s) (e.g. risk ratio, mean difference) used in the synthesis or presentation of results. | Page 7 |
| Synthesis methods | 13a | Describe the processes used to decide which studies were eligible for each synthesis (e.g. tabulating the study intervention characteristics and comparing against the planned groups for each synthesis (item #5)). | Page 7 |
|  | 13b | Describe any methods required to prepare the data for presentation or synthesis, such as handling of missing summary statistics, or data conversions. | Page 7 |
|  | 13c | Describe any methods used to tabulate or visually display results of individual studies and syntheses. | Page 6-7 |
|  | 13d | Describe any methods used to synthesize results and provide a rationale for the choice(s). If meta-analysis was performed, describe the model(s), method(s) to identify the presence and extent of statistical heterogeneity, and software package(s) used. | Page 8 |
|  | 13e | Describe any methods used to explore possible causes of heterogeneity among study results (e.g. subgroup analysis, meta-regression). | Page 8 |
|  | 13f | Describe any sensitivity analyses conducted to assess robustness of the synthesized results. | Page 8 |
| Reporting bias assessment | 14 | Describe any methods used to assess risk of bias due to missing results in a synthesis (arising from reporting biases). | Page 8 |
| Certainty assessment | 15 | Describe any methods used to assess certainty (or confidence) in the body of evidence for an outcome. | Page 8 |
| **RESULTS** | | |  |
| Study selection | 16a | Describe the results of the search and selection process, from the number of records identified in the search to the number of studies included in the review, ideally using a flow diagram. | Page 9 (figure 1) |
|  | 16b | Cite studies that might appear to meet the inclusion criteria, but which were excluded, and explain why they were excluded. | Page 9 (figure 1) |
| Study characteristics | 17 | Cite each included study and present its characteristics. | Page 9 (table 1) |
| Risk of bias in studies | 18 | Present assessments of risk of bias for each included study. | Page 10 (Supplementary table S2) |
| Results of individual studies | 19 | For all outcomes, present, for each study: (a) summary statistics for each group (where appropriate) and (b) an effect estimate and its precision (e.g. confidence/credible interval), ideally using structured tables or plots. | Page 10-11 (Table 1 and 2) |
| Results of syntheses | 20a | For each synthesis, briefly summarise the characteristics and risk of bias among contributing studies. | Page 11-12 (Supplementary table S2) |
|  | 20b | Present results of all statistical syntheses conducted. If meta-analysis was done, present for each the summary estimate and its precision (e.g. confidence/credible interval) and measures of statistical heterogeneity. If comparing groups, describe the direction of the effect. | Page 11-12 (Figure 2 and 3) |
|  | 20c | Present results of all investigations of possible causes of heterogeneity among study results. | Page 11-12 (Supplementary table S4 and supplementary figure S1-4) |
|  | 20d | Present results of all sensitivity analyses conducted to assess the robustness of the synthesized results. | Page 11-12 (Supplementary table S4 and supplementary figures S1-4) |
| Reporting biases | 21 | Present assessments of risk of bias due to missing results (arising from reporting biases) for each synthesis assessed. | Page 12 (Figure 4) |
| Certainty of evidence | 22 | Present assessments of certainty (or confidence) in the body of evidence for each outcome assessed. | Page 12 (Figure 4) |
| **DISCUSSION** | | |  |
| Discussion | 23a | Provide a general interpretation of the results in the context of other evidence. | Page 12-17 |
|  | 23b | Discuss any limitations of the evidence included in the review. | Page 17-18 |
|  | 23c | Discuss any limitations of the review processes used. | Page 17-18 |
|  | 23d | Discuss implications of the results for practice, policy, and future research. | Page 17-18 |
| **OTHER INFORMATION** | | |  |
| Registration and protocol | 24a | Provide registration information for the review, including register name and registration number, or state that the review was not registered. | Abstract  Page 5 |
|  | 24b | Indicate where the review protocol can be accessed, or state that a protocol was not prepared. | Abstract  Page 5 |
|  | 24c | Describe and explain any amendments to information provided at registration or in the protocol. | Page 5 |
| Support | 25 | Describe sources of financial or non-financial support for the review, and the role of the funders or sponsors in the review. | NA |
| Competing interests | 26 | Declare any competing interests of review authors. | NA |
| Availability of data, code and other materials | 27 | Report which of the following are publicly available and where they can be found: template data collection forms; data extracted from included studies; data used for all analyses; analytic code; any other materials used in the review. | NA |

**.**

**Supplementary Table S9**: Original study protocol

| **Project title** | Association between ultra-processed food intake and cognitive impairment: a systematic review and meta-analysis |
| --- | --- |
| **First reviewer** | Dr xxx |
| **Second reviewer** | Dr xxx |
| **Supervisor** | Professor xxx |

| 1. **Background to review** |
| --- |
| Cognitive impairment exists on a spectrum from mild cognitive impairment (MCI) to dementia; a chronic and progressive syndrome that presents with deterioration in memory and cognition beyond what would be expected in the normal physiological ageing process; impacting upon activities of daily life (ADLs) [2]. 60-70% of cases are attributed to Alzheimer’s disease (AD), although other conditions exist under the dementia umbrella including vascular, frontotemporal and Lewy body dementia [3]. Owing to ageing populations globally, due to the advancement of health care, dementia prevalence is increasing. It is now the most common disease in the elderly, with over 55 million cases reported worldwide according to World Health Organisation (WHO) statistics. Annually, the incidence of dementia is at a rate of 10 million new cases, and so by 2050 it is estimated that 140 million people will be living with dementia [2]. At present, there is no cure for dementia, and as such research focuses on areas that could be targeted to help prevent and delay the disease burden. One area that is emerging in scientific literature is the association between dementia and metabolic syndrome (MetS); with risk of dementia increasing for every additional component of the MetS present [4]. Socioeconomic status is a known predictor of MetS [5], and it has also be shown to predict risk of dementia [6], suggesting that health inequalities may be in part culpable for the increasing dementia prevalence.  A significant pathophysiological driver of the burgeoning metabolic disease prevalence, and the increasing disparities and health inequalities seen among contrasting socioeconomic groups, is a dramatic transformation in the global food system with rapid growth of ultra-processed food (UPF) consumption [7]. UPFs are industrial formulations of cheap ingredients from high yield crops (such as refined sugar, starch, oil, protein isolates) and remnants of intense animal agriculture that are highly energy-dense due to total fat, saturated fat and trans-fat contributions, combined with low fibre and poor micronutrient profiles [8]. They include such food items as cookies, confectionary sweets, high-sugar drinks and ‘microwave ready-meals’, constituting around half of total daily energy intake in Western populations [9]. Their cheap production cost, contrasting with the higher relative cost of minimally processed foods, drives a high UPF consumption rate globally; particularly in low income households [10]. A further exacerbating factor is that their consumption promotes a vicious cycle by further increasing caloric intake and progressive weight gain [11]. A myriad of food processing classification systems have been developed to assess the processing level of food. However, a recent systematic review highlighted NOVA as the most specific, coherent, clear, comprehensive and workable of these systems [12].  To date, meta-analyses have demonstrated positive associations between diet quality and dementia, with adherence to a Mediterranean diet, high in polyunsaturated fatty acids (PUFA) and vegetables, being protective [13, 14]. Meta-analyses have also demonstrated association between UPF and various non-communicable diseases such as components of the MetS including overweight and obesity, type 2 diabetes (T2D), non-alcoholic fatty liver disease (NAFLD), cardiovascular disease (CVD) and cancer risk [15-18]. In addition, experimental study highlights probable associations between UPF intake and dementia [19-21]. However, meta-analysis has not yet objectively explored the association between UPF and dementia.  Thus, the primary aim of the current review is to assess and quantify the relationship between consumption of UPF and the prevalence of dementia through systematic review and meta-analysis. The secondary aim is to assess whether a dose-response relationship exists between UPF consumption and dementia. |
| 1. **Specific objectives** |
| - To assess and quantify the relationship between UPF and MCI and dementia through systematic review of existing literature - To assess and quantify the relationship between consumption of different types of dementia - To assess the dose-response effect of the relationship between UPF and dementia through meta-analysis |

| 1. **Inclusion criteria – PICOS** | |
| --- | --- |
| **Population** | - Adult patients with MCI or dementia (age 18 years or older) - Dementia can include Alzheimer disease, vascular dementia, frontotemporal dementia and Lewy body dementia, but not dementia of familial or inherited aetiology, or cognitive decline associated with genetic syndromes - Any gender - Any race - Not restricted to UK but paper needs to be written in English |
| **Intervention/exposure** | - UPF consumption - Classified by NOVA system directly or indirectly - Assessed through dietary assessment tools |
| **Control** | Diets that are more minimally processed |
| **Outcome** | 1. Prevalence of MCI or all-cause dementia in patients with higher UPF intake 2. Prevalence of different dementia dementia in patients with higher UPF intake 3. Dose-response relationship between UPF intake and prevalence of MCI and dementia |
| **Study design** | - Must define the outcome of interest as MCI or dementia based on clearly stated diagnostic criteria or identified through diagnostic codes with additional confirmation. This should include use of formal assessment of cognitive function or dementia or clear implication that formal dementia diagnosis took place (eg, cognitive decline assessed using general screening or neuropsychological testing, dementia diagnosis using standard diagnostic tools) - Observational studies (including prospective or retrospective cohort studies, case-control and cross-sectional study designs) |

| 1. **Exclusion criteria** |
| --- |
| - Animal studies - In vitro studies - Secondary research including other review articles - Paediatric population (<18 years of age) - Studies that only focused on beverages - Case reports, editorials, abstracts, unpublished studies or practice guidelines - Duplicates - Study that reports cases of familial or inherited dementia, or cognitive decline in association with any genetic syndrome - Study reporting outcomes as delirium |

| 1. **Search methods** | |
| --- | --- |
| **Electronic databases** | Ovid Medline, Web of Science |
| **Other methods** | Reference checking  Contacting experts in field |

| 1. **Review methodology** | |
| --- | --- |
| **Details of methods** | Two reviewers (AH and CG) will use the previously defined inclusion and exclusion criteria to select appropriate literature from Medline and Web of Science. There will be three stages to the selection process. Firstly, articles will be screened by titles by the two reviewers independently of each other. The second stage involves reading of the study abstracts that make it through title screening. Finally, full texts of articles that make it through title and abstract screening will be read. Any disagreements will be resolved via discussion. In addition, the authors will perform manual searches of reference lists of relevant studies and contacted experts in the field to identify any other articles not already identified. If an expert thought a currently unidentified paper was suitable for inclusion, this will be discussed via video call with the first reviewer (AH). |
| **Quality assessment** | Each selected article will have its quality of evidence independently scrutinised through use of the Newcastle Ottawa scale (NOS) [22] by both reviewers (AH and CG). As per a recent meta-analysis comparing the association between UPF and T2DM, we will stratify evidence into three groups: poor quality research scored <5 stars, medium quality research scored 5 or 6 stars and high-quality research scored >6 stars [15].  In addition, the NutriGrade scoring system will be used to assess the overall credibility of evidence. The tool is an eight-item scale that evaluates evidence for meta-analyses related to nutrition specifically. To interpret NutriGrade evaluation, the following scoring system will be used: a) very low (0–3.99); b) low (4–5.99); c) moderate (6–7.99); d) high (8–10) [23]. |
| **Data extraction** | Rayyan will be used to store and extract data.  From the included studies, the following data will be extracted for use in the current systematic review and meta-analysis by reviewer one (AH) and checked by reviewer two (CG): **1)** basic study information (author name, year of publication, journal) and **2)** study design (population, country, study type, sample size, follow up, adjustment for confounding variables, definitions used for UPF and dementia (including dietary assessment tool, whether the study evaluated UPFs by NOVA classification system directly or not, diagnostic tool for dementia), study outcomes (including reported risk estimates in relation to dementia development (OR, HR or RR and 95% confidence intervals)). For studies that did not report the necessary data, efforts were made to contact the corresponding author. |
| **Systematic review** | Narrative synthesis will be done alongside meta-analysis and will be carried out using a framework which consists of four elements:   - Developing a theory of how the exposure works, why and for whom - Developing a preliminary synthesis of findings of included studies - Exploring relationships within and between studies - Assessing the robustness of the synthesis |
| **Meta-analysis** | A random effects model will be used to calculate a pooled RR ± 95% CI for all the included studies due to expecting high heterogeneity to exist. This will be conducted using R Studio software. The Higgins I^2^ statistical technique will be used to assess heterogeneity between the included studies [24]. To be considered highly heterogenous, values will be required to be above 75% with a p value <0.05. Furthermore, meta-regression will be performed to help generate a dose-response graph.  Funnel plots will be generated an, if there are over ten included articles, Eggers test will be performed to quantify publication bias.  Finally, sensitivity analyses will be performed using random or fixed effects models to see whether results would change dependent on study design (longitudinal or non-longitudinal), reporting of UPF (NOVA or non-NOVA classified), sample size (<10,000 or >10,000 participants), continent (North America, Europe or Asia), type of dementia, and study quality based on NOS (to help determine whether high risk of bias impacted upon outcomes). This will be done to evaluate whether a single, or several, study or studies disproportionately affected the overall effect size. |
| **Scoring evidence** | Newcastle Ottawa Scale  NutriGrade tool |

| 1. **Presentation of results** | |
| --- | --- |
| **Additional material** | - Protocol - Flow chart of whole process   Data extraction form and tables   - Funnel plot to visually highlight publication bias - Forest plots of studies included in the final review - Meta-regression bubble plot to demonstrate dose-response relationship between UPF intake and dementia |
| **Outputs** | - 1 paper in high quality obesity journal. This include submission to any of the following: **1)** Age and Ageing, **2)** Journal of Neurology, Neurosurgery and Psychiatry, or **3)** Neurology - Conference presentations |

| 1. **Timeline** | |
| --- | --- |
| **Protocol** | Week 1 |
| **Literature search** | Week 3 |
| **Quality appraisal** | Week 4 |
| **Data extraction** | Week 5 |
| **Synthesis** | Week 7 |
| **Writing up** | Week 9 |
